# Community-Engaged Research: Common Themes and Needs Identified by Investigators and Research Teams at an Emerging Academic Learning Health System

**DOI:** 10.3390/ijerph18083893

**Published:** 2021-04-08

**Authors:** Megan B. Irby, Keena R. Moore, Lilli Mann-Jackson, DeWanna Hamlin, Isaiah Randall, Phillip Summers, Joseph A. Skelton, Stephanie S. Daniel, Scott D. Rhodes

**Affiliations:** 1 Maya Angelou Center for Health Equity, Wake Forest School of Medicine, Winston-Salem, NC 27157, USA; mirby@wakehealth.edu; 2 Program in Community-Engaged Research, Wake Forest School of Medicine, Winston-Salem, NC 27157, USA; krmoore@wakehealth.edu (K.R.M.); irandall@wakehealth.edu (I.R.); 3 Department of Social Sciences and Health Policy and Program in Community-Engaged Research, Wake Forest School of Medicine, Winston-Salem, NC 27157, USA; lmann@wakehealth.edu; 4 Formerly of the Program in Community-Engaged Research, Wake Forest School of Medicine, Winston-Salem, NC 27157, USA; dlthamlin@yahoo.com; 5 Department of Radiology, Wake Forest School of Medicine, Winston-Salem, NC 27157, USA; psummers@wakehealth.edu; 6 Department of Pediatrics and Program in Community-Engaged Research, Wake Forest School of Medicine, Winston-Salem, NC 27157, USA; jskelton@wakehealth.edu; 7 Department of Family and Community Medicine and Program in Community-Engaged Research, Wake Forest School of Medicine, Winston-Salem, NC 27157, USA; sdaniel@wakehealth.edu

**Keywords:** community-engaged research, health disparities, academic learning health system, qualitative methods

## Abstract

Community-engaged research (CEnR) has emerged within public health and medicine as an approach to research designed to increase health equity, reduce health disparities, and improve community and population health. We sought to understand how CEnR has been conducted and to identify needs to support CEnR within an emerging academic learning health system (aLHS). We conducted individual semi-structured interviews with investigators experienced in CEnR at an emerging aLHS in the southeastern United States. Eighteen investigators (16 faculty and 2 research associates) were identified, provided consent, and completed interviews. Half of participants were women; 61% were full professors of varied academic backgrounds and departments. Interviews were audio-recorded, transcribed, coded, and analyzed using constant comparison, an approach to grounded theory. Twenty themes emerged that were categorized into six domains: Conceptualization and Purpose, Value and Investment, Community-Academic Partnerships, Sustainability, Facilitators, and Challenges. Results also identified eight emerging needs necessary to enhance CEnR within aLHSs. The results provide insights into how CEnR approaches can be harnessed within aLHSs to build and nurture community-academic partnerships, inform research and institutional priorities, and improve community and population health. Findings can be used to guide the incorporation of CEnR within aLHSs.

## 1. Introduction

### 1.1. Community-Engaged Research

Community-engaged research (CEnR) has emerged within public health and medicine as an approach to research designed to increase health equity, reduce health disparities, and improve community and population health. CEnR involves the affected community in research, and defines that community as any group of people affiliated by geographic proximity, special interest, health condition, or similar categories of shared identity. Rather than investigators and research teams from universities, government, or other types of research organizations approaching and entering a community with a preconceived notion of a community’s best interests, in projects that apply CEnR approaches, community members and representatives from community organizations collaborate and share research roles with academic investigators and research teams. Community members become not merely “targets” of research but also research partners. CEnR emphasizes relationship-building and trust; open communication; co-learning; reciprocal transfer of expertise; shared power, resources, and decision-making; and mutual ownership of the processes and products of research [1,2,3,4,5,6,7,8,9].

Growing evidence suggests that including community members and representatives from community organizations in the design, implementation, and evaluation of research can lead to deeper, more informed, and nuanced understandings of health-related phenomena and identify actions (e.g., interventions, programs, and policy and system changes) that are more relevant, culturally congruent, and likely to be effective, sustained, and scalable, if warranted, to improve community and population health [6,7,9,10,11]. However, the conduct of CEnR is shaped by institutional (e.g., allocation of resources and time), community (e.g., local history and receptivity), and personal (e.g., background, experiences, and world views) factors. Thus, there is no standardized way to engage and partner with communities to conduct CEnR, and there is great variability across models of engagement and in the degree to which communities are included in the research process [8,9].

Community engagement often is viewed as a continuum that spans from outreach (less engaged), consultation, involvement, and collaboration, to shared leadership (more engaged) [3,4,7,12,13]. As such, the community engagement literature is extensive and features a wide range of theories, approaches, strategies, and methods, reflecting tremendous diversity in how community engagement is defined, implemented, and evaluated [1,2,6,7,13]. Despite this heterogeneity, CEnR includes several common critical elements. These include: (1) Collaboration with groups of people affiliated by geographic proximity, special interest, health condition, or other categories of shared identity; (2) Groups of community members, organizational representatives, and academic researchers adhering to common principles and norms to nurture trust and promote authentic partnership; (3) Focus on identifying and addressing the needs and priorities and harnessing the assets that affect health and well-being; and (4) Research as an approach to systematically uncover and understand health-related phenomena and improve community and population health [2,3,7].

### 1.2. Academic Learning Health Systems

Learning health systems (LHSs) are organizations or networks that pursue a cycle of perpetual learning processes embedded in daily practice. These processes inform evidence-based improvements in health care to yield better patient outcomes. This cycle combines quality improvement methods and data analytics from patient care, which are fed back into the LHS to inform institutional policies and structures and improve care delivery [14]. An academic learning health system (aLHS) prioritizes gathering new, generalizable knowledge to improve community and population health, in addition to promoting continuous improvement and innovation within its own health care delivery system. An aLHS draws on its embedded academic expertise in public health and medical education, health system sciences, translation, and population and community health; shares knowledge and imparts skills necessary to advance the system internally; and disseminates knowledge broadly to contribute to knowledge generation and advance science [15,16].

Although engaging patients to provide perspectives and feedback into various aspects of health systems has long been valued, to date, there has been no exploration of CEnR within aLHSs. This is due in part to the recent emergence of the aLHS as a concept and entity [15,16,17]. Understanding how CEnR has been conducted and identifying needs to support and enhance CEnR within an emerging aLHS could provide insights critical to advance our knowledge of effective CEnR methods and designs and strengthen the conduct of future research designed to increase health equity, reduce health disparities, and improve community and population health [18].

Wake Forest School of Medicine/Wake Forest Baptist Health (WFSM/WFBH), an emerging aLHS, has a long and successful history of CEnR [19,20,21,22,23]. A number of its investigators routinely incorporate community engagement in their research. We sought to explore how CEnR has been harnessed at WFSM/WFBH; how investigators at this emerging aLHS have incorporated principles of community engagement within their research; the degree to which community members and/or representatives from community organizations have been involved in the planning, implementation, evaluation, and/or dissemination phases of research; the nature of CEnR as it is implemented within the context of the local community; and what institutional supports are needed to facilitate and integrate CEnR within an aLHS.

## 2. Materials and Methods

### 2.1. Setting

WFSM/WFBH is an emerging aLHS serving more than 50 counties in North Carolina, Tennessee, Virginia, and West Virginia. It is comprised of WFSM and five hospitals in the Piedmont region of northwest North Carolina. In addition to training medical, physician assistant, and nursing students, WFSM has a broad array of biomedical graduate and postdoctoral training programs. It also has a large Division of Public Health Sciences with Departments in Biostatistics and Data Science, Epidemiology and Prevention, Implementation Science, and Social Sciences and Health Policy. A Clinical and Translational Science Award (CTSA) from the National Center for Advancing Translational Sciences (NCATS), National Institutes of Health (NIH), supports the WFSM/WFBH Clinical and Translational Science Institute (CTSI), which includes the Program in Community-Engaged Research (UL1TR001420). The Program aims to improve community and population health through promoting and facilitating community-relevant and engaged research, working in partnership with communities to identify health needs, priorities, and assets and to seek solutions to health challenges together [23].

### 2.2. Participants

We conducted individual semi-structured interviews with investigators experienced in community engagement and CEnR. We identified potential interviewees with assistance from the WFSM/WFBH CTSI and Institutional Review Board (IRB). A keyword search was performed within the institutional grants management software (InfoEd) and IRB databases to identify WFSM/WFBH investigators who had submitted research applications related to community engagement or CEnR within the past ten years. Search terms included “community”, “engagement”, “CBPR”, “participatory research”, and “partnership”. Abstracts from identified applications were reviewed to further determine eligibility. Investigators were then contacted by electronic mail and invited to participate in an interview. Interviews with consenting participants were then scheduled at a time and location convenient to the participant. Interviews were conducted by two trained study staff and digitally recorded, with study staff also taking clarifying notes. During interviews, participants were asked for the names of other investigators within the institution who also conducted CEnR to identify additional potential participants. Interviews were transcribed verbatim and subsequently verified.

### 2.3. Individual Semi-Structured Interview Guide

We chose a qualitative research approach to gain a broad spectrum of perspectives about CEnR within the context of an aLHS. This qualitative approach can yield perspectives and insights that are not previously known by researchers and thus would not emerge in a more close-ended quantitative approach [24,25]. A semi-structured interview guide was designed to collect both descriptive demographic data from each participant, including race/ethnicity, age, gender, position, academic degrees, background, current academic department, years at WFSM/WFBH, and CEnR training. The guide also captured socio-contextual and detailed descriptions of perceptions, experiences, and strategies specific to the use of community engagement and CEnR. Areas explored included: how each participant conceptualizes and defines CEnR; their preparation for CEnR, including formal training; perceived benefits of CEnR; challenges associated with CEnR; how communities are engaged in research; what roles community members and representatives from community organizations play in CEnR; how the region (i.e., southern United States) and the institution (i.e., an emerging aLHS) affect CEnR; how sustainability and dissemination of CEnR are incorporated into CEnR; and what CEnR lessons have been learned by each participant. The guide is summarized in Table 1.

The semi-structured interview guide was drafted, reviewed, revised, and finalized by community members with experience in community-engaged research and experts in community engagement and aLHSs. All items were open-ended. The guide was pilot tested for comprehension and timing with 3 investigators; slight revisions to wording were made based on the pilot.

### 2.4. Analysis

Each interview transcript was coded by two analysts. Themes were identified through constant comparison, an approach to developing grounded theory, combining inductive coding with simultaneous comparison [25]. Using standard procedures [25], analysts first coded text and convened to compare their codes. They then identified and resolved any discrepancies through discussion. Matrices were used to identify similarities and differences within and across participants. Analysts identified, refined, and interpreted themes iteratively through discussion and by examining codes and rereading the transcripts. Findings and themes were presented to interview participants in a presentation for the WFSM/WFBH CTSI Program in Community-Engaged Research Affinity Group (*n* = 27). The Affinity Group is a group of investigators, research team members, and others at WFSM/WFBH who are interested in CEnR. Members of the Community Stakeholder Advisory Committee (CSAC) (*n* = 8) of the CTSI’s Program in Community-Engaged Research also attended. CSAC is comprised of representatives from community organizations who provide feedback to the CTSI regarding research infrastructure and policies, and to investigators and research teams regarding WFSM/WFBH research initiatives [26] Presentation attendees contributed to the refinement of themes and their interpretation through facilitated group discussion.

Human subject approval and oversight for this study were provided by the WFSM/WFBH IRB.

## 3. Results

The keyword search in InfoEd and IRB databases yielded the names and research projects of 51 investigators. Sixteen investigators were confirmed as eligible based on a review of project abstracts; 14 of these responded to email invitations and agreed to participate. Four additional participants were referred by initial participants. In all, 18 investigators (16 faculty and 2 senior research associates) provided consent and completed interviews. Interviews averaged 45 min.

The participant sample (Table 2) was 50% female, had a mean age of 55 years, was mostly White, and had varied academic backgrounds. Most participants held doctoral degrees (i.e., PhD, DrPH, EdD, MD, and MD/PhD). On average, participants had worked at WFSM/WFBH for nearly 14 years, and represented six academic departments. Nearly all participants indicated they had never received formal education or training in CEnR, though 100% reported “on-the-job” training and experience. All had been principal investigators on at least one federally funded research project.

### 3.1. Domains and Themes Related to Community Engagement and CEnR

Twenty themes emerged across six domains related to community engagement and CEnR within an aLHS (Table 3): Conceptualization and Purpose, Value and Investment, Community-Academic Partnerships, Sustainability, Facilitators, and Challenges.

#### 3.1.1. Conceptualization and Purpose of CEnR

Participants agreed that CEnR is a collaborative approach to research designed to improve health and well-being through participatory and better-informed inquiry, always with an eye on how knowledge generated can be translated and applied within the local affected community. However, participants noted that this local application of knowledge does not preclude its generalizability and transferability to other contexts, thus aligning with the broader research goals of an aLHS. Participants also identified CEnR as an approach relevant within many aspects of an aLHS, including education and training, quality improvement, clinical care, and clinical trials.

Participants highlighted the difficulties in defining “community” and emphasized that communities are heterogeneous, which can be challenging for investigators and research teams who may want “simple answers” (e.g., perspectives and insights) from community partners; as participants reported, working with one community does not yield one voice. CEnR was identified as requiring careful consideration of the various perspectives and insights of all partners. Finally, participants also identified additional goals of CEnR, including strengthening connections within the community, building resilience and capacity, and reducing the effects of marginalization.

#### 3.1.2. Value and Investment in CEnR: Institutional, Professional, and Personal

Participants described CEnR as an under-appreciated and frequently misunderstood approach to research within many institutions, including federal funding agencies such as the NIH, the U.S. Centers for Disease Control and Prevention (CDC), and the Health Resources and Services Administration (HRSA). A participant reported that this misunderstanding among federal partners persists even when a project is required by the federal partner and funder and initially designed to be conducted using CEnR approaches. A participant provided an example of an intervention study that resulted in null findings; the participant attributed the study’s null findings to funder-required changes to the study design, recruitment and retention strategies, and an intervention that did not align with community partner perspectives and were contrary to project-specific community steering committee guidance.

Participants also noted that their professional and academic investment in CEnR stemmed from their own personal values, including community health, health equity, and social justice. Participants agreed that this commitment was not limited to traditional nine-to-five “work hours,” but that successful community engagement must be woven into daily life and interactions to be successful.

#### 3.1.3. Community-Academic Partnerships

Participants expressed that the type and degree of engagement, and the ease with which partnerships are formed, depend on the extent to which communities feel they have been marginalized and how they perceive research and research institutions. Moreover, partnerships, how they function, and the roles of partnership members in CEnR vary profoundly across investigators and across projects. Participants identified many areas as essential to the development of strong and productive partnerships: fostering trust and mutual respect, balancing expertise across community and aLHS partners, investing time, facilitating open communication, embracing conflict as a strategy for resolution, and overcoming barriers (including addressing community mistrust based on previous community experiences with both research and health care). Participants noted the role of the Tuskegee Syphilis Study [27], the Guatemalan Syphilis Experiments [28]), and rampant anti-immigration rhetoric [29], racism, homophobia, and transphobia in the United States as contributing to community mistrust.

#### 3.1.4. Sustainability of CEnR

Participants described sustaining CEnR as complex, citing both the need and the difficulty of maintaining community-aLHS partnerships beyond the time period of an individual project. They highlighted the difficulty in sustaining partnerships without funding for continued community involvement and effort from academic investigators and research teams. Additionally, participants noted that sustainability is influenced by the strength and quality of engagement throughout a project, and how well community partners were incorporated into the research process—from conception, study design and conduct, data analysis and interpretation, to the dissemination of findings.

#### 3.1.5. Facilitators of CEnR

Participants emphasized the need for institutional support (e.g., funding, protected time, and respect) and research resources (e.g., CTSA and institutional research centers) that prioritize CEnR as integral to the academic mission of an aLHS. Participants also noted the immense value of having an IRB that is willing to learn the nuances of CEnR in order to approve and oversee CEnR.

#### 3.1.6. Challenges of CEnR

Participants described the potential for burn out and strain resulting from the need to incorporate community engagement into daily life and the great time commitment accompanying CEnR. Other challenges identified by participants related to the discordance between community and academic goals within a given research project, and differences in overarching community and academic priorities that challenged collaboration. Participants also described the challenges of history and how an institution’s complicity in oppressive practices of the past influenced the development and maintenance of community research partnerships. For example, some participants cited WFSM/WFBH’s past involvement in the North Carolina Eugenics Program [30] as reducing community trust and engagement.

### 3.2. Enhancing Community-Engaged Research within an aLHS

We also identified eight emergent needs that could enhance CEnR within aLHSs (Table 4). First, participants described the need for increased understanding among academic investigators, research teams, and healthcare providers of community contexts and assets, social determinants of health (also known as “social drivers of health”), and historical factors that influence community and population health. Participants reported that this increased understanding may be particularly critical because many investigators, research teams, and providers may be from other regions of the country or other parts of the world. Thus, although well intentioned, they may not sufficiently understand the local community or the contexts of the populations of interest and focus.

Similarly, participants noted a profound need for increased understanding of CEnR within the aLHS and its value as an approach within community and population health, public health, and medicine. Participants expressed frustration that CEnR is often conflated with community outreach; formative or qualitative research; or behavioral and social sciences. Participants emphasized that members of communities have critical perspectives regarding research into locally identified needs and priorities. Such collaborations can harness the assets of both the community and the aLHS.

Third, participants also highlighted the need for training community members, academic investigators, and research teams to increase their understanding of and skills in partnering with communities and conducting CEnR. Participants noted that establishing authentic and productive partnerships to conduct CEnR is difficult, and, despite good intentions, many investigators and research teams at the emerging aLHS do not understand how to work effectively with community members and representatives from community organizations. At the same time, participants shared that community partners may not sufficiently understand the research process, how evidence and knowledge are generated, and the various components of and objectives inherent within an aLHS.

Fourth, participants noted that although the theories and principles underlying CEnR are well documented, effective frameworks and methods aligned with CEnR are needed. They noted the need for methodologic innovations. Participants noted methods such as photovoice [31], empowerment-based community forums [32], evidence academies [33], and citizen science [23] are well developed, further research approaches to and methods aligned with CEnR are needed.

Participants also noted that policy changes can positively affect health in multiple ways, whether within an aLHS, the local community, or nationally. They identified a need for evidence-based, practical guidance to increase the translation of CEnR findings into policies designed to improve community and population health.

Sixth, participants acknowledged the need for guidance on balancing the perspectives of community and aLHS partners. They reported that it can be difficult for academic investigators and research teams to know how to elicit community partner perspectives, to usefully share their own perspectives (based on theory, their own prior research, and the existing literature), build on and negotiate with community partners, and negotiate and compromise in ways that ensure sound science and maximize the success of a research project. Participants noted that CEnR requires weighing scientific rigor and what is realistic and “doable”; far too often, participants reported, investigators and research teams may choose rigorous research approaches that simply cannot be successfully implemented. Participants asserted not including community perspectives through CEnR could result in study designs that are inauthentic to how communities convene, interact, and take action; enrollment and retention plans that are not acceptable or realistic; and/or measurement that does not make sense to members of the community. In such situations, data collection may be sacrificed, analysis and interpretation of findings may be less accurate, and sustainability and meaningful dissemination of findings may not be possible.

Participants cited a need for a model to incorporate principles of CEnR into research mission, vision, and priorities of an aLHS. They suggested that the linkages between CEnR and aLHS need further exploration and articulation. Finally, participants reported the need for more institutional support for community engagement and CEnR. This support included pilot funding and protected time of investigators and research teams to establish partnerships, develop innovative methods, and explore integration of community perspectives into the priorities and processes of an aLHS.

## 4. Discussion

Our results provide insights critical to understanding how CEnR approaches function within an emerging aLHS and ways to further build and nurture community-academic partnerships and inform research and institutional priorities to increase health equity, reduce health disparities, and improve community and population health. In this study, we identified six primary domains of 20 themes related to the purpose of CEnR, its value within an aLHS, characteristics of effective community-aLHS partnerships, issues related to sustainability of CEnR, facilitators of CEnR within an aLHS, and challenges facing CEnR within an aLHS. Many of these domains have been explored in the broader CEnR literature; however, this is the first exploration of CEnR within an aLHS. We also uncovered eight needs that, if addressed, could support and enhance community engagement and CEnR within an aLHS. Several findings deserve highlighting.

First, participants expressed the need for academic investigators, research teams, and healthcare providers to better understand community contexts, social determinants of health, and historical factors influencing community and population health and participation in research. This view is consistent with previous research suggesting that health disparities will persist without better understanding of health and health-related phenomena within communities and the ongoing reluctance of community members to participate in research [11].

Participants also described the importance of the aLHS’s reputation in the community as critical to influencing trust and engagement, particularly among historically marginalized populations. Participants specifically noted WFSM/WFBH’s past involvement in the North Carolina Eugenics Program and other discriminatory practices committed against minority and vulnerable populations [30]; they also noted that many communities across the United States share similar historical narratives. Thus, although much work must done; the inclusion of CEnR within CTSAs and emerging aLHS is a step in the right direction.

Trust is built by and exists among individuals; community members may or may not trust an institution, but the ongoing commitment of investigators and research teams from an aLHS to partner with and listen to community members can overcome mistrust [21]. Thus, thorough training for academic investigators and research teams is essential. This training should include how to work collaboratively; how to encourage, elicit, and listen to diverse voices; and how to help partners organize for community and population health. While training in designing studies, reducing bias, and increasing validity are critical for investigators and research teams, skills in relationship building and maintenance and in negotiation and compromise are similarly critical [2,11,34,35]. For example, when investigators and research teams from an aLHS attend community fairs, church gatherings, community forums, or parties and celebrations, these informal settings help build and nurture trust among partners. These opportunities show commitment and allow attendees to further understand one another. Volunteering with a community organization or serving on local health coalitions are other ways to advance trust and develops genuine and mutually respectful relationships between researchers and communities. In addition, this involvement can open other doors by helping to identify others in the community who may be committed to working together [21].

Nearly all participants in this study highlighted the need for institutions to explicitly show their value of CEnR. This finding aligns with previous work that identified barriers attributable to institutional culture that shape research agendas and support for CEnR, and challenges in gaining support from institution leadership and top decision-makers [8,11]. Overcoming these challenges and cultural aspects specific to aLHSs likely requires leadership to further explore institutional readiness to serve as a partner to communities, the existence and appropriateness of structures to support CEnR scholarship, investigator and study staff training in CEnR, the extent to which aLHS goals align with principles of CEnR, and whether there is sufficient CEnR expertise within the institution to establish a commitment to CEnR [11,34,35]. Participants echoed the need for CEnR training opportunities (for investigators, research teams, and community members); education on effective models of engagement across diverse populations; resources to conduct CEnR authentically; and guidance for interpreting findings and disseminating information back to communities.

This study was conducted at a single aLHS; thus, findings may not be applicable to other aLHSs. Regardless, the study’s design and analysis may help other aLHSs and medical centers, as well as those seeking to incorporate CEnR approaches within public health and medicine, to engage and form authentic and long-lasting partnerships with communities. The sample size achieved saturation across interviews and provides valuable information regarding the number of investigators and research teams conducting CEnR. In this study, we did not collect data from community research partners, which was beyond the scope of our work. Future research is warranted to better understand perspectives of community members and community organizations with or without previous experience partnering with investigators and research teams within aLHSs.

## 5. Conclusions

While some findings from this study reflect the broader CEnR literature, these findings are important for informing CEnR approaches and can be used to guide the incorporation of CEnR within aLHSs. LHSs and aLHSs are becoming more established and numerous, and many institutions could benefit from our findings. In educating the next generation of academic investigators, research teams, and healthcare providers, an aLHS can incorporate CEnR as it strives to understand and increase health equity, reduce health disparities, and improve community and population health.

## Figures and Tables

**Table 1:** Abbreviated items from the individual semi-structured interview guide.

Abbreviated Items from the Individual Semi-Structured Interview Guide.
How do you define community engagement and CEnR?
2. What do you consider to be some of the defining characteristics of CEnR?
3. Tell me about the experiences, personal or professional, that prepared you for the CEnR that you are part of.
4. What are the some of the benefits of conducting CEnR?
5. What are the some of the challenges of and barriers to conducting CEnR?
6. Who from the community is typically involved in the research you are part of, and in what ways? How do you engage communities in research and the research process? What kinds of different roles do community members have throughout the research process?
7. I’m interested in your perspectives on conducting CEnR in the southeastern United States and within an aLHS. Share with me experiences you feel are unique to this region and institution, as well as those that are experienced more broadly. How might your experiences and research projects differ compared to other regions and at other institutions? To what can you attribute these differences and/or similarities? What impact, if any, does being at an emerging aLHS have on your approach to CEnR?
8. Tell me about the dissemination of findings from the CEnR that you are part of and about your perspectives on sustainability. How are findings disseminated? How is the research you are part of sustained? Where do resources come from to support sustainability? How do your expectations of funding influence your research and the projects you choose to pursue?
9. What lessons have you learned from your experiences that you would want to share with them?

**Table 2:** Demographics of participants (*N* = 18).

Demographics	*n* (%) *
**Race/Ethnicity**	
Black	2 (11%)
White	16 (89%)
**Age (mean)**	55 years
**Gender**	
Female	9 (50%)
Male	9 (50%)
**Professional Rank**	
Research Associate	2 (11%)
Assistant Professor	2 (11%)
Associate Professor	3 (17%)
Professor	11 (61%)
**Terminal Degree**	
Bachelors	1 (6%)
Masters	1 (6%)
PhD, DrPH, EdD	13 (72%)
MD	2 (11%)
MD-PhD	1 (6%)
**Academic Background**	
Anthropology	2 (11%)
Education	1 (6%)
Exercise Science	1 (6%)
Medicine	3 (17%)
Neuroscience	1 (5%)
Psychology	4 (22%)
Public Health	4 (22%)
Public Policy	1 (6%)
Sociology	1 (6%)
**Academic Department**	
Dermatology	1 (6%)
Epidemiology & Prevention	5 (28%)
Family Medicine	3 (17%)
Neurology	1 (6%)
Social Sciences & Health Policy	7 (39%)
Psychiatry	1 (6%)
**Years at WFSM/WFBH (mean)**	4–27 years (13.9)
**Level of Training in Community Engagement or CEnR**	
Formal Training	2 (11%)
No Formal Training	16 (89%)
On-the-Job Training	18 (100%)

* Count and percent or mean and standard deviation.

**Table 3:** Domains and themes related to conducting community-engaged research (CEnR) at an academic learning health system (aLHS).

Domains	Themes
Conceptualization and Purpose of CEnR	CEnR is a collaborative approach to research with translational impact that is both local and generalizable. CEnR can be implemented across the many facets of an aLHS. “Community” often is difficult to define. CEnR is meant to strengthen connections within the community, build resilience and capacity, and reduce the effects of marginalization while improving community and population health.
Value and Investment in CEnR: Institutional, Professional, and Personal	CEnR is underappreciated within and not well understood by institutions and by major funding agencies (e.g., NIH and CDC). Community engagement is of personal and professional value, which strengthens commitment to authentic engagement.
Community-Academic Partnerships	Engagement differs by community and is influenced by the extent to which communities feel marginalized and how communities perceive research, investigators, and institutions. Partnerships, how they function, and the roles partnership members play in CEnR vary across investigators and across projects. Partnerships must value, respect, and learn how to harness and balance the expertise of the community and the academic partners. CEnR takes time. Forming community-academic partnerships may require “relationship repair” to overcome barriers associated with mistrust and/or previous harms committed in the name of research.
Sustainability of CEnR	Partnerships should not end when the funding ends. Achieving sustainability can be difficult. Sustainability is influenced by the strength and quality of engagement throughout a project, and how well community partners were incorporated into various phases of the research process.
Facilitators of CEnR	Institutional support enhances the ability to conduct CEnR by providing funding, protected time, and respect for community engagement as a valid and important approach to research and health care. Having research resources such as an NIH Clinical and Translational Science Award (CTSA) reinforces CENR as integral to the academic mission. An Institutional Review Board that understands and is willing to learn the nuances of CEnR approaches.
Challenges of CEnR	Burn out and strain are possible. Differences in community and academic priorities and goals can impede CEnR efforts, strain relationships, and make it more difficult to collaborate. Institutional and community history influence how well partnerships can be formed and maintained.

**Table 4:** Emergent needs to support and enhance community-engaged research (CEnR) within an academic learning health system (aLHS).

Emergent Needs to Support and Enhance Community-Engaged Research (CEnR) within an Academic Learning Health System (aLHS).
Increased understanding among investigators, research teams, and healthcare providers of community contexts and assets, social determinants of health, and historical factors that influence community and population health
2. Enhanced understanding of CEnR and its value as an approach within community and population health, public health, and medicine
3. Training opportunities for community members, investigators, and research teams to increase their understanding of and skills in partnering with communities and conducting rigorous CEnR
4. Identification of effective frameworks and methods aligned with CEnR
5. Evidence-based practices to translate CEnR findings into policy change
6. Guidance for balancing the perspectives of community and academic partners
7. A model for incorporating CEnR into the mission, vision, and priorities of an aLHS
8. Institutional support for community engagement and CEnR

## Data Availability

Data are available from the corresponding author.
